# Intravenous Iron–Induced Hypophosphatemia in Surgical Patients

**DOI:** 10.1001/jamanetworkopen.2025.3093

**Published:** 2025-04-17

**Authors:** Toby Richards, Linda K. Wijaya, Jayne Lim, Cory Dugan, Darren Dahly

**Affiliations:** 1 School of Health, Sport & Bioscience, University of East London, London, United Kingdom; 2 Institute of Clinic Trials and Methodology, University College London, London, United Kingdom; 3 Department of Anaesthesia & Perioperative Medicine, Monash University, Melbourne, Australia; 4 Faculty of Medicine, Nursing and Health Sciences, Monash University, Melbourne, Australia; 5 Telethon Kids Institute, Perth, Australia; 6 UWA Medical School, University of Western Australia; 7 University College Cork, Cork, Ireland

## Abstract

This study explores the incidence of preoperative hypophosphatemia and whether hypophosphatemia may have affected patient or trial outcomes for those who received ferric carboxymaltose.

## Introduction

Intravenous (IV) iron has become a standard treatment for iron deficiency and anemia. Modern formulations of IV iron enable a large dose to be administered safely with a low complication rate comparable to other infusions.^1^ However, ferric carboxymaltose (FCM) can cause hypophosphatemia mediated by fibroblast growth factor 23 (FGF23) that increases urinary excretion of bone phosphate.^2^ While this is often an asymptomatic laboratory finding, concern has arisen from reports of osteomalacia and bone fractures.^3^

PREVENTT was a multicenter randomized clinical trial that compared IV FCM (1000 mg; n = 244) to placebo (saline; n = 243) to treat anemia before major open abdominal surgery.^4^ As the main clinical end points of blood transfusion, death, or perioperative complications were not different between the groups in the main trial, we hypothesized that if FCM caused hypophosphatemia, it was clinically asymptomatic. The aim of this study was to explore the incidence of preoperative hypophosphatemia in PREVENTT and, in the cohort of patients who received FCM, whether hypophosphatemia may have affected patient or trial outcomes.

## Methods

Blood samples were taken as part of the PREVENTT trial protocol at randomization (421 of 487 patients) and on the day of surgery (392 of 487 patients) from January 6, 2014, to September 28, 2018 (eAppendix in Supplement 1). Samples were only analyzed after trial completion so local sites and patients were blinded to results.

Additional information about laboratory analyses and the main trial data can be found in the eMethods in Supplement 1. Hypophosphatemia was defined as normal (>0.8 mmol/L), mild (0.65-0.8 mmol/L), and moderate or severe (<0.65 mmol/L).

We also conducted an exploratory analysis of data from patients in the FCM cohort, describing differences in baseline characteristics and key study outcomes by phosphate status (<0.65 vs ≥0.65 mmol/L). For this cohort analysis, threshold of *P* < .05 indicated statistical significance.

The PREVENTT trial received ethical approval from the UK National Research Ethics Committee, East of England, and all patients gave informed consent. Reporting of this study followed the STROBE reporting guideline.

## Results

In PREVENTT, IV FCM given a median of 14 days (IQR, 12-21 days) before major abdominal surgery significantly reduced preoperative serum phosphate levels (−0.21 mmol/L; 95% CI, −0.27 to −0.14 mmol/L; *P* < .001; Figure). This was associated with increased intact FGF23 levels (mean difference, 30.3 pg/mL; 95% CI, 23.1 to 37.5 pg/mL; *P* < .001).

**Figure.**
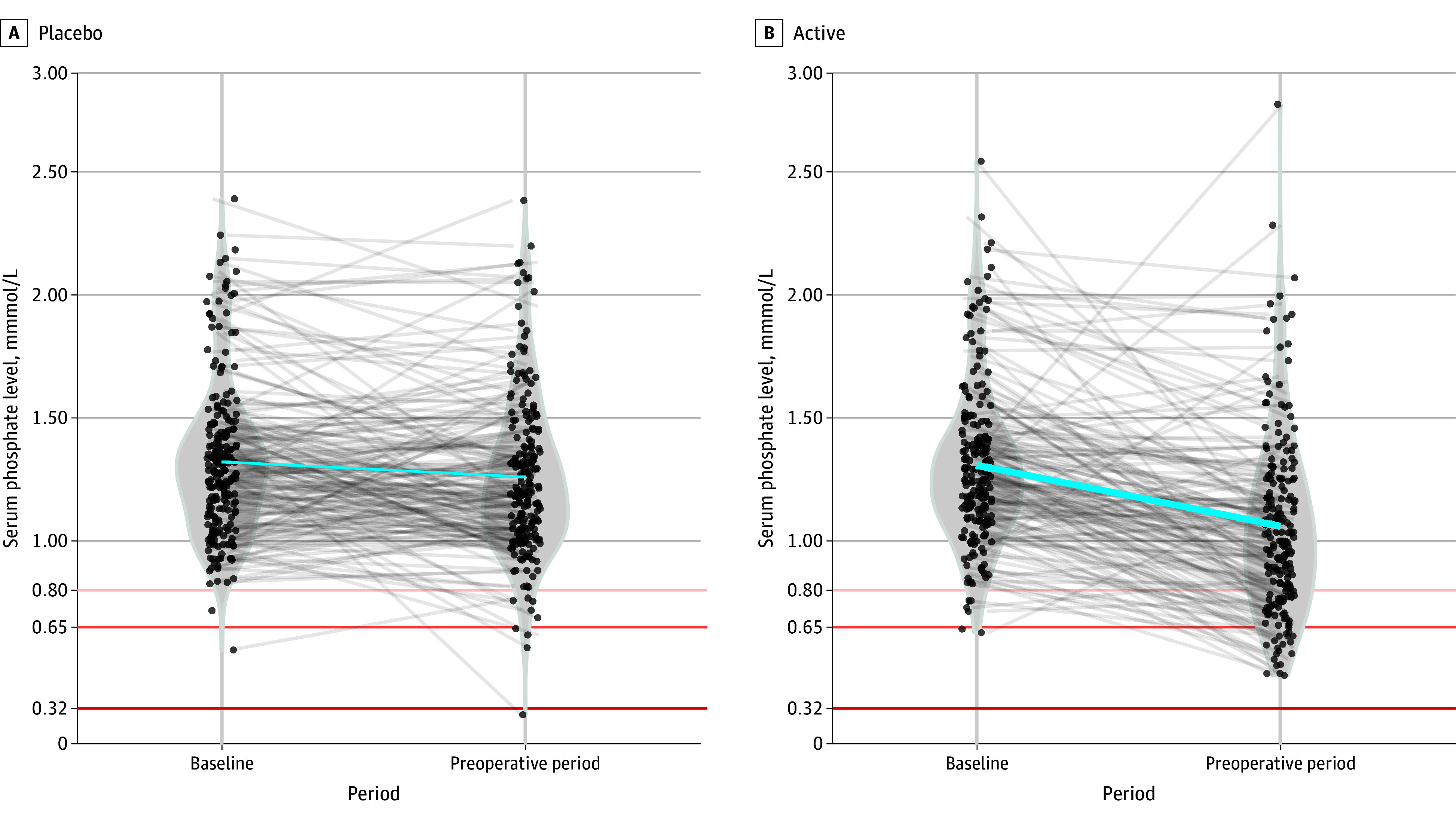
Changes in Serum Phosphate Levels From Baseline to Preoperative Period in Placebo and Ferric Carboxymaltose (FCM) Groups This figure displays the changes in serum phosphate levels from baseline to the preoperative period for both the placebo (A) and FCM treatment (B) groups. Each black dot represents an individual participant’s serum phosphate level at baseline and the preoperative period. The gray lines connect the baseline and preoperative values for each participant, with a mean line (blue) showing the trend of serum phosphate changes within individuals. The horizontal lines indicate critical thresholds of serum phosphate levels: tan indicates moderate hypophosphatemia at <0.80 mmol/L; orange, mild at <0.65 mmol/L; and red, severe at <0.32 mmol/L.

Patients receiving FCM were more likely to develop hypophosphatemia (<0.8 mmol/L) (51 of 194 [26%]) compared to placebo (9 of 198 [4.5%]) (odds ratio, 9.22; 95% CI, 4.45-21.2; *P* < .001). There were no patient characteristics associated with increased risk of FCM-induced hypophosphatemia (Table).

**Table.** Outcomes Among Active Arm Participants (FCM Group), Comparing Those With Post-Iron Hypophosphatemia (<0.65 mmol/L) vs Those Without

Characteristic	Patients treated with FCM, No. (%)		
	Total (N = 194)	Phosphate levels, mmol/L	
		≥0.65 (n = 174)	<0.65 (n = 20)
Sex			
Female	103 (53)	94 (54)	9 (45)
Male	91 (47)	80 (46)	11 (55)
Age, median (IQR), y	67 (56-72)	67 (57-72)	69 (55-76)
Had surgery	193 (99)	173 (99)	20 (100)
Days between iron and surgery, median (IQR)	14 (12-21)	15 (13-21)	13 (11-14)
Surgery type			
AAA	1 (<1)	1 (1)	0
Colorectal	26 (13)	20 (12)	6 (30)
General	17 (9)	14 (8)	3 (15)
Gynaecological	55 (28)	52 (30)	3 (15)
Upper GI	70 (36)	64 (37)	6 (30)
Urological	24 (12)	23 (13)	2 (10)
ASA grade			
I	24 (13)	22 (13)	2 (11)
II	120 (65)	108 (64)	12 (67)
III	41 (22)	38 (23)	3 (17)
IV	1 (<1)	0	1 (6)
V	0	0	0
Iron status			
Normal	38 (21)	36 (22)	2 (11)
Functional iron deficiency	89 (49)	80 (49)	9 (47)
Absolute iron deficiency	56 (31)	48 (29)	8 (42)
Outcomes			
Hemoglobin concentration, median (IQR), g/L	113 (102-120)	112 (102-120)	114 (110-120)
BT or death within 30 d of surgery (PREVENTT primary outcome)	56 (29)	49 (28)	7 (35)
Length of ITU stay, median (IQR), d	2 (0-3)	2 (0-3)	2 (0-6)
Length of hospital stay, median (IQR), d	10 (7-14)	9 (6-14)	12 (10-22)^a^
No. of days alive and out of hospital up to 30 d postsurgery, median (IQR)	21 (16-24)	22 (17-24)	19 (10-22)^a^

Abbreviations: AAA, abdominal aortic aneurysm; ASA, American Society of Anesthesiologists; BT, blood transfusion; FCM, ferric carboxymaltose; GI, gastrointestinal tract; ITU, intensive therapy unit.

^a^ Indicates significant difference (*P* < .05) between groups (Pearson χ^2^ test; Wilcoxon rank sum test; Fisher exact test).

FCM-induced hypophosphatemia was associated with increased median hospital stay (12 [IQR, 10-22] days vs 9 [IQR, 6-14] days; *P* = .01) and reduced median days alive and out of hospital (19 [IQR, 10-22] days vs 22 [17-24] days; *P* = .02) (Table). Secondary analysis of the FCM cohort suggested that FCM-induced moderate or severe hypophosphatemia (20 of 194) was associated with increased rates of postoperative adverse events (11 of 20 [55%] vs 44 of 174 [26%]; *P* = .008).

## Discussion

In PREVENTT, patients randomized to FCM had a 9-fold increased risk of preoperative hypophosphatemia, which was associated with increased length of hospital stay and reduced days alive and out of hospital. Complications in PREVENTT were assessed by the Clavien-Dindo classification, and routine phosphate levels not recorded. As such, like other trials on IV iron, a limitation was that adverse event monitoring may not have had the granularity to detect the (often vague) symptoms of hypophosphatemia, such as muscle weakness or pain, fatigue, or confusion, which could have been overlooked or normalized after major surgery.

In a cohort study^5^ of women receiving FCM, no association between infusion adverse events and phosphate levels was seen. However, in the PHOSPHARE-IBD study, decreases in phosphate concentration were associated with changes in Fatigue Scale scores.^6^ As with PREVENTT, these data suggest that FCM-induced hypophosphatemia may be a problem in some patient groups (inflammatory bowel disease and surgery).

With increased use of IV iron, there is increased awareness on the issue of hypophosphatemia, which is particularly a concern as generic FCMs become available (with less clinical trial data available). This clinical concern is exacerbated by the lack of consensus on if or how to treat FGF23-mediated hypophosphatemia, as treatment with phosphate supplementation can be counterproductive due to increased parathyroid hormone that may worsen FGF23-mediated urinary phosphate excretion. FCM-induced hypophosphatemia may be a problem that requires further investigation, as in patients before major surgery it may be symptomatic and associated with longer hospital stay.

## Notes

Supplement 1.
eMethods.

eAppendix.

Supplement 2.
Data Sharing Statement
