# Supplementary material for: Intravenous Iron–Induced Hypophosphatemia in Surgical Patients
Source: JAMA Netw Open. 2025 Apr 17;8(4):e253093. doi: 10.1001/jamanetworkopen.2025.3093 (PMC12006868; doi:10.1001/jamanetworkopen.2025.3093)
Supplement: Supplement 1. — eMethods. eAppendix. [file jamanetwopen-e253093-s001.pdf]

Supplemental Online Content

Richards T, Dugan CW, Wijaya L, Lim J, Dahly DL. Intravenous iron–induced hypophosphatemia in surgical patients: a secondary analysis of the PREVENTT randomized clinical trial. *JAMA Netw Open*. 2025;8(4):e253093.
doi:10.1001/jamanetworkopen.2025.3093

eMethods.

eAppendix. Adverse Events Reported For Those With and Without Hypophosphatemia

This supplemental material has been provided by the authors to give readers additional information about their work.

© 2025 Richards T et al. *JAMA Network Open*.

eMethods.

Blood samples were taken as part of the PREVENTT trial protocol at randomization (421/487) and on the day of surgery (392/487) from Jan 6, 2014, to Sept 28, 2018. (Supplementary file). Samples were only analysed after trial completion so local sites and patients were blinded to results.

Laboratory analyses included a full blood count, iron studies, serum phosphate, and intact FGF23 immunoassays (Kainos FGF-23 ELISA Kit (CY-4000), undertaken in 2022.

For the main trial data a complete-case, intention-to-treat analysis of 355 patients using baseline-adjusted regression was used to confirm the impact of FCM treatment (vs placebo) on preoperative phosphate levels, odds of hypophosphatemia, and intact FGF23 levels.

© 2025 Richards T et al. *JAMA Network Open*.

eAppendix. Adverse Events Reported For Those With and Without Hypophosphatemia

Adverse events reported for

PV43001	Postoperative ileus
PV12008	Anal fissure
PV12008	Abdominal wound dehiscence
PV12010	Vomiting
PV12010	Abdominal hernia repair
PV14007	Abdominal pain
PV14012	Auditory hallucinations
PV32017	Clostridium difficile infection
PV33004	Nausea
PV33004	Nausea
PV33004	Cephalgia
PV33004	Hypoalbuminemia
PV33004	Abdominal pain
PV33004	APTT prolonged: PT prolonged
PV33004	Wound infection
PV33004	Malaise
PV41008	Headache: Dizzy
PV41008	Nausea: Vomiting
PV42004	Constipation
PV42004	Wound infection
PV42004	Vomiting
PV44003	Alanine aminotransferase increased
PV44003	Shoulder pain
PV44003	Pyrexia
PV44003	Phosphate low
PV59013	Pneumothorax
PV59013	Arterial catheterisation abnormal
PV59013	Low blood pressure

© 2025 Richards T et al. *JAMA Network Open*.

those with hypophosphatemia

	Adverse events reported for those without hypophosphatemia
PV11001	Renal replacement therapy
PV11002	Anastomotic leak
PV12002	Nausea: Vomiting
PV12002	Postoperative ileus: Postoperative infection
PV12018	Wound infection
PV14089	Nosocomial pneumonia
PV16010	Abdominal pain
PV16010	Constipation
PV18009	Fall
PV25008	Haematemesis
PV43003	Postoperative ileus
PV44009	Collapse of lung
PV56001	Chest infection
PV56002	Paralytic ileus
PV58003	Postoperative ileus
PV59007	Localised intraabdominal fluid collection
PV59007	Urinary retention postoperative
PV59018	Respiratory failure
PV67003	Postoperative bleeding
PV10008	Itchy
PV10008	Alkaline phosphatase increased
PV10008	Feeling of body temperature change
PV10022	Drug-induced headache
PV11002	Pulmonary oedema
PV12002	Localised itching: Skin scaly
PV12005	Hay fever
PV12006	Pain in hip
PV12006	Chipped tooth
PV12006	Vomiting
PV12014	Diarrhoea

© 2025 Richards T et al. *JAMA Network Open*.

PV12014	Drug-induced delirium
PV12022	Upper respiratory tract infection
PV13001	Blood test abnormal
PV14001	Hypertension
PV14010	Blood pressure high
PV14022	Diarrhoea
PV16003	Toe injury
PV16004	Back pain
PV16004	Dizzy spells
PV16004	Vaginal bleeding
PV16010	Wound infection
PV18003	Blood in urine
PV18004	Wound infection
PV18004	Aggressive behaviour: Unresponsive to verbal stimuli
PV18004	Ventricular ectopics
PV18009	Blood test abnormal
PV18009	Hypoglycaemia
PV18009	Stomach pain
PV18009	Oxygen saturation decreased
PV18009	Confusion
PV21004	Lethargy
PV21004	Headache
PV21004	Dizziness
PV21004	Chickenpox
PV23001	Headache
PV25001	Pins and needles
PV41006	Overdose
PV42010	Infusion related reaction
PV43007	Nosocomial pneumonia
PV43007	Atrial fibrillation
PV44002	Phosphate low
PV44002	Pyrexial
PV44002	Infusion site discolouration

PV44002	Haemoglobin low
PV44002	Headache
PV44009	Atrial fibrillation
PV44009	Abnormal sensation of limbs
PV44009	Chest pain
PV45007	Rash
PV47004	Hypoalbuminaemia
PV47004	Nosocomial pneumonia
PV47004	Atrial fibrillation
PV48003	Pelvic sepsis
PV48003	Small bowel obstruction
PV48005	Ear infection
PV48006	Chylothorax
PV52001	Atrial fibrillation
PV56002	Hypophosphataemia
PV56004	Phosphate low
PV59007	Hallucinations
PV59007	Wound infection
PV59007	Localised intraabdominal fluid collection
PV59007	Oedema legs
PV59007	Postoperative constipation
PV59009	Wound complication
PV59018	Postoperative wound complication
PV59018	Leg oedema
PV67001	Intraoperative splenic injury

© 2025 Richards T et al. *JAMA Network Open*.
