# Supplementary material for: Intravenous Iron–Induced Hypophosphatemia in Surgical Patients
Source: JAMA Netw Open. 2025 Apr 17;8(4):e253093. doi: 10.1001/jamanetworkopen.2025.3093 (PMC12006868; doi:10.1001/jamanetworkopen.2025.3093)
Supplement: Supplement 2. — Data Sharing Statement [file jamanetwopen-e253093-s002.pdf]

Data Sharing Statement

Richards. Intravenous Iron–Induced Hypophosphatemia in Surgical Patients. *JAMA Netw Open*. Published April 10, 2025. doi:10.1001/jamanetworkopen.2025.3093

Data
Data available: Yes
Data types: Deidentified participant data
How to access data: toby.richards@ucl.ac.uk
When available: With publication

Supporting Documents
Document types: None

Additional Information
Who can access the data: Anyone is welcome to present a proposal to the PEVENTT team for data sharing
Types of analyses: Any
Mechanisms of data availability: UCL requires a data sharing agreement
Research data and other material (study protocol, statistical analysis plan, informed consent form) will be available to the scientific community, immediately on publication, with as few restrictions as possible (researchers whose proposed use of the data has been approved). All requests should be submitted to the corresponding author for consideration. Researchers who provide a methodologically sound proposal likely to maximise the value of the data for patient and public benefit will be approved (subject to review by a subgroup of the authors).
